# Impaired emotion processing in functional (psychogenic) tremor: A functional magnetic resonance imaging study

**DOI:** 10.1016/j.nicl.2017.10.020

**Published:** 2017-10-18

**Authors:** Alberto J. Espay, Thomas Maloney, Jennifer Vannest, Matthew M. Norris, James C. Eliassen, Erin Neefus, Jane B. Allendorfer, Anthony E. Lang, Jerzy P. Szaflarski

**Affiliations:** a Gardner Family Center for Parkinson's Disease and Movement Disorders, Cincinnati, OH, USA; b Pediatric Neuroimaging Research Consortium, Cincinnati Children's Hospital, Cincinnati, OH, USA; c University of Cincinnati Center for Imaging Research (CIR), Cincinnati, OH, USA; d Department of Neurology, University of Alabama at Birmingham, Birmingham, AL, USA; e The Morton and Gloria Shulman Movement Disorders Clinic and the Edmond J. Safra Program in Parkinson's Disease, University Health Network, University of Toronto, Toronto, Canada.

**Keywords:** AFNI, Analysis of Functional Neuroimages, CPT-END, continuous performance task with emotional and neutral distracters, FSL, FMRIB Software Library, FT, functional tremor, fMRI, functional magnetic resonance imaging, HAM-A, Hamilton Anxiety Rating Scale, HAM-D, Hamilton Depression Rating Scale, EPI, echo-planar imaging, MDEFT, modified equilibrium Fourier transform, MINI, Mini International Neuropsychiatric Interview, Functional tremor, Psychogenic tremor, Functional movement disorders, Conversion disorder, fMRI, Emotion processing

## Abstract

**Background:**

Despite its high prevalence and associated disability, the neural correlates of emotion processing in patients with functional (psychogenic) tremor (FT), the most common functional movement disorder, remain poorly understood.

**Methods:**

In this cross sectional functional magnetic resonance imaging (fMRI) study at 4T, 27 subjects with FT, 16 with essential tremor (ET), and 25 healthy controls (HCs) underwent a finger-tapping motor task, a basic-emotion task, and an intense-emotion task to probe motor and emotion circuitries. Anatomical and functional MRI data were processed with FSL (FMRIB Software Library) and AFNI (Analysis of Functional Neuroimages), followed by seed-to-seed connectivity analyses using anatomical regions defined from the Harvard-Oxford subcortical atlas; all analyses were corrected for multiple comparisons.

**Results:**

After controlling for depression scores and correcting for multiple comparisons, the FT group showed increased activation in the right cerebellum compared to ET during the motor task; and increased activation in the paracingulate gyrus and left Heschl's gyrus compared with HC with decreased activation in the right precentral gyrus compared with ET during the basic-emotion task. No significant differences were found after adjusting for multiple comparisons during the intense-emotion task but increase in connectivity between the left amygdala and left middle frontal gyrus survived corrections in the FT subjects during this task, compared to HC.

**Conclusions:**

In response to emotional stimuli, functional tremor is associated with alterations in activation and functional connectivity in networks involved in emotion processing and theory of mind. These findings may be relevant to the pathophysiology of functional movement disorders.

## Highlights

• Patients with functional tremor exhibit altered emotion processing circuitry.
• There is increased activation in the paracingulate gyrus after emotional stimuli.
• Increased connectivity between the left amygdala and middle frontal gyrus
• Functional disorders may be associated with disturbances in the theory of mind.

## 1 Introduction

Functional (psychogenic) tremor (FT), the most common functional movement disorder, is diagnosed by confirming entrainment or full suppressibility of the oscillatory activity, distractibility, co-activation or co-contraction sign, pause of tremor during contralateral ballistic movements, and variability in tremor frequency, axis, and/or topographical distribution (Espay and Lang, 2015). Despite its frequency and the magnitude of disability it imparts, the pathophysiological underpinnings of FT remain poorly understood and no effective treatments have been established.

Neuroimaging studies have suggested that the basal ganglia and limbic systems are integral parts of the neural pathways for processing emotions (Nowak and Fink, 2009). Recent functional neuroimaging studies of patients with functional movement disorders have demonstrated alterations in regional cerebral blood flow during simple motor tasks (Schrag et al., 2013) or in brain activation of the cerebellar vermis, posterior cingulate cortex, and hippocampus on isometric precision-grip contraction tasks (Blakemore et al., 2016) as well as in brain activation of the right amygdala on simple emotional stimuli (n = 10) (Voon et al., 2010) or both amygdala on stimulation on fearful emotional stimuli (n = 12) (Aybek et al., 2015). We hypothesized that patients with FT have an impairment and/or disconnection of cortical and subcortical areas involved in motor and emotion control that may be distinguished from those of essential tremor (ET) and healthy controls (HCs). This hypothesis is also complemented by preliminary findings of differences in emotion processing in other neurological disorders (Allendorfer and Szaflarski, 2014, Szaflarski et al., 2014). ET is the most common tremor disorder, diagnosed in the presence of postural and action hand tremor, often in the context of a positive family history. While it follows none of the diagnostic criteria for FT, ET is considered to represent cerebellar dysfunction although with poorly defined neurobiological boundaries (Espay et al., 2017). We chose basic and intense emotion processing fMRI tasks in order to access the emotional state of the observed individual and as a measure of social intelligence, a concept separate from general (or cognitive) intelligence (Bar-On et al., 2003, Bonora et al., 2011). Further, the brain regions responsible for facial emotion recognition and processing, which includes visual (spatial cognition) and executive (attentional control) networks may be involved directly or indirectly in the generation or maintenance of FT (Calarge et al., 2003).

## 2 Methods

### 2.1 Subjects

Twenty-seven consecutive consenting patients with FT met established clinical criteria (Espay and Lang, 2015, Fahn and Williams, 1988, Gupta and Lang, 2009). Tremor needed to be absent or minimal at rest in order to avoid interference with the scanning procedure. Patients were excluded if they had any comorbid neurological disorder or severe depression or anxiety as measured by a Hamilton Depression Rating Scale (HAM-D) > 24 and a Hamilton Anxiety Rating Scale (HAM-A) > 25. Subjects were also excluded if they were on benzodiazepines for any reason. We also prospectively recruited 16 consecutive patients with ET as active controls (given the common misdiagnosis of FT with ET and vice versa), and 25 HCs with no history of neurological or general medical conditions. This study was approved by the local IRB and all subjects provided informed consent.

To reach the subject goal of 27 FT patients completing all assessments and rendering high-quality dataset for analyses, we screened a total of 35 FT subjects. Eight screened subjects, 6 with FT and 3 with ET were not recruited due to the following reasons (one each for FT, unless otherwise specified): malingering (rather than conversion), not meeting criteria for FT, patient unwilling to provide consent, unacceptance of diagnosis, prior neurosurgical procedure (unable to undergo fMRI), excessive tremor during scanning, obesity beyond scanner's capacity (1 ET), and inability to get comfortable in scanner (2 ET, 2 FT). Data from 10 subjects was excluded from final analysis due to non-completion of the task or for data quality issues: basic-emotion processing task: 1 HC; intense-emotion processing task: 1 FT, 1 HC; VBM analysis: 4 HCs, 1 ET; volumetric analysis: 2 HCs.

### 2.2 Clinical measurements

All subjects underwent a 15-minute structured diagnostic interview (Mini International Neuropsychiatric Interview; MINI) (Pinninti et al., 2003) developed to screen for axis I DSM-IV and ICD-10 psychiatric disorders (Sheehan et al., 1998). In addition, we administered the 17-item HAM-D (Williams, 1988) to assess depressed mood and vegetative and cognitive symptoms of depression; and the 14-item HAM-A (Maier et al., 1988), to evaluate for psychic and somatic anxiety. These scales were administered as part of a structured interview (Williams et al., 2008).

### 2.3 Functional MRI procedure

Anatomical and functional brain images were obtained using a 4T MRI/MRS system (Varian Inc.). The behavioral experiment was programmed in E-Prime, version 1 (www.pstnet.com). All participants wore MR-compatible VGA goggles and headphones (Resonance Technologies, Inc.). For each imaging session, once the participant was positioned in the scanner, a three-plane scout scan was performed to confirm isocenter positioning prior to each of the functional tasks. An echo-planar imaging (EPI) was performed while subjects carried out the behavioral paradigms using a T2*-weighted gradient-echo EPI pulse sequence: TR/TE 3000/29 ms, FOV 256 × 256 mm, matrix 64 × 64, slice thickness 4 mm, flip angle 75°. A multi-echo reference scan was performed to correct for geometric distortion and Nyquist ghost artifacts. After completion of all functional MRI (fMRI) tasks a T1-weighted three-dimensional anatomical high-resolution scan using modified equilibrium Fourier transform (MDEFT) sequence (TR/TE 13/6 ms, T(MD) 1.1 s, FOV 192 × 256 × 256 mm, matrix 192 × 256 × 265, slice thickness 1 mm, flip angle 20°) was acquired (Lee et al., 1995). The MRI system triggered the behavioral paradigms to ensure precise timing of the task with respect to image acquisition.

### 2.4 Imaging paradigms

Three paradigms were used during the functional scans to examine differences in motor and emotional processing between the three groups. The paradigms, a finger-tapping motor task, a basic-emotion task, and an intense-emotion task were presented to each participant in the same order.

*Finger-tapping motor task* was designed to assess and monitor the motor system while in the scanner. This paced task consisted of a 30-second block of right-only finger tapping, followed by a 30-second block of left-only tapping, followed by a 30-second block of rest, all repeated 4 times. Subjects were instructed to adhere to the provided rate with the visual prompt presented every second. The task required subjects to move a lever using their right or left index finger, according to whether the “R” or “L” was flashing. The total task duration was 6 min. Task adherence was monitored visually. The task was modeled such that the blocks of rest were treated as “baseline” in the analyses.

The “*basic-emotion*” *face recognition task* was designed to assess response to basic emotional stimuli. Over the span of 14 min, subjects were presented with 120 different faces, corresponding to unique (non-repeating) facial identities each depicting a particular emotion (sadness, happiness, or fear) or a neutral expression (Szaflarski et al., 2014). Processing of emotional expressions is thought to occur subliminally and automatically but is dependent on attention (Pessoa et al., 2002b, Rees et al., 1997). To monitor attention to the task, subjects were instructed to decide the gender of each face by pressing one of two buttons with the right thumb. Subjects were exposed to 30 prototypically happy, 30 sad, 30 fearful and 30 neutral expressions presented in random order selected from the NimStim set of facial expressions (Tottenham et al., 2009). Each stimulus was presented for 2 s with variable inter-stimulus interval of 3.9 ± 2.4 s; during the delay subjects viewed a fixation cross. Subjects were asked to press button “1” for males and button “2” for females while viewing each image. It is well recognized that activation by faces in some brain areas is strongly affected by attentional condition while in other brain areas it is not (e.g., amygdala response to fearful stimuli) (Bentley et al., 2003, Pessoa et al., 2002a). The event-related design with variable inter-stimulus delay was used to reduce habituation of the activation in regions such as the amygdala, since habituation may occur in block designs with highly repetitive and predictable stimulus presentation (Breiter et al., 1996).

The “*intense-emotion*” *task* (continuous performance task with emotional and neutral distracters; CPT-END) consisted of a series of offensive or disgusting images probing intense emotional circuitry (Yamasaki et al., 2002). This task utilized a visual oddball paradigm where 70% of the cues were squares, 10% were circles (targets), 10% were emotionally unpleasant pictures, and 10% were emotionally neutral pictures. Subjects held the same response box as for the basic-emotion task and were asked to press with the right thumb a “2” for circles and “1” for all other images. There were two runs of the task in the imaging session. There were 158 total cues with 3 s per cue and a constant display time of 2.75 s with a 0.25 s interval with fixation cross. Emotional and neutral pictures originated from the International Affective Picture System (University of Florida, Gainesville, Florida) and were selected based on criteria previously utilized (Strakowski et al., 2011).

### 2.5 Image processing and statistical analysis

Reconstruction of the raw data was performed with 3D Hamming filter using in-house software developed in IDL (www.ittvis.com) (Schmithorst et al., 2001). First-level fMRI data processing was carried out using FSL (FMRIB Software Library) (Jenkinson et al., 2012, Smith et al., 2004) and AFNI (Analysis of Functional Neuroimages) (Cox, 1996).

### 2.6 Anatomical data

Data were first reoriented to standard orientation using FSL's fslreorient2std. Next, the T1 data were bias corrected and brain extracted using FSL's FAST(Zhang et al., 2001) and BET respectively (Smith, 2002). The brain extracted image was then normalized to the 2 mm isotropic MNI ICBM 152 non-linear 6th Generation template (Grabner et al., 2006) using FSL's FLIRT (Jenkinson et al., 2002, Jenkinson and Smith, 2001). Subcortical segmentation was performed using FSL's FIRST (Patenaude et al., 2011) on the bias corrected image in native space.

### 2.7 Functional data

Typical pre-processing steps, such as reorientation, slice timing correction and brain extraction, were carried out using FSL's fslreorient2std, slicetimer and BET (Smith, 2002), respectively. Outlying functional volumes, based on motion and intensity, were detected using FSL's fsl_motion_outliers. Motion correction of the BOLD time-series was carried out using MCFLIRT (Jenkinson et al., 2002). The functional file was interpolated to 2 × 2 × 2 mm voxel size and aligned to the Montreal Neurological Institute (MNI) template (Grabner et al., 2006) by first co-registering it with the participant's T1 using FSL's FLIRT (Jenkinson et al., 2002, Jenkinson and Smith, 2001). The motion related artifacts were then regressed from the data by setting up a general linear model design using 24 motion parameters (6 motion parameters, the 6 motion parameters squared, a first order autoregressive model of the 6 motion parameters and a first order autoregressive model of the 6 motion parameters squared) plus an additional parameter for each detected outlier (Friston et al., 1996). A CompCor regression was also implemented using the eigenvectors from the first five out of ten principle components within a white matter and CSF mask respectively (Behzadi et al., 2007). The residuals from the GLM were high-pass filtered in accordance with the task timing, 0.008 Hz for finger tapping and 0.04 Hz for CPT and faces, and smoothed with a 6 mm FWHM filter using AFNI's 3dBandpass. All task-based group results were corrected for multiple comparisons using FSL's threshold free cluster enhancement (TFCE) (Smith and Nichols, 2009), a non-parametric permutation test, with 5000 iterations. Each of the functional group comparisons was modeled both, with and without the HAM-D score as a covariate of interest. While measures of both anxiety and depression were collected, they are highly correlated, so therefore we chose to use only HAM-D scores in our analysis.

### 2.8 Seed-to-seed connectivity analysis

**Fig. 1:**
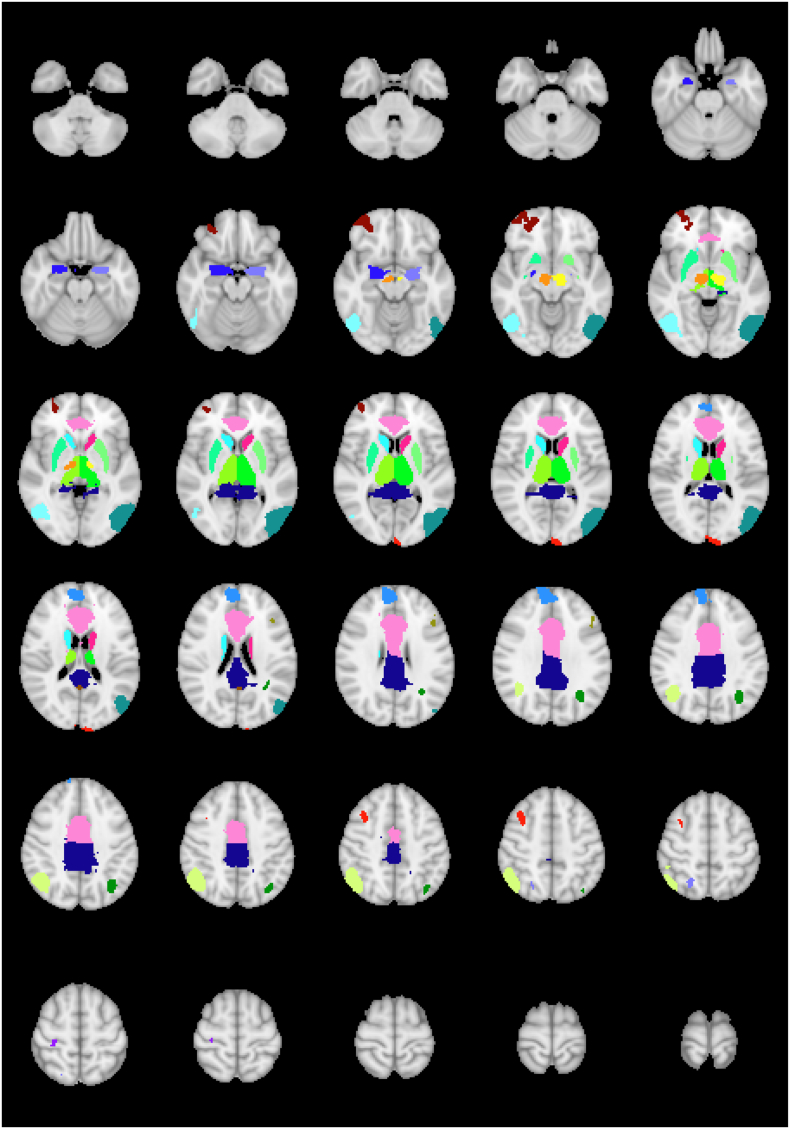
Regions of interest used in connectivity analysis based on the activation patterns from the intense-emotion task and subcortical atlas. The anatomical regions of interest included the anterior cingulate gyrus, posterior cingulate gyrus and left and right regions of the uncus/amygdala, thalamus, caudate, putamen, and subthalamic nuclei. The regions from the group activation of the task included regions in the left cerebral cortex, right frontal pole, right postcentral gyrus, left precuneus cortex and right superior frontal gyrus along with two regions in the left lateral occipital cortex, two regions in the left cingulate gyrus, three regions in the right lateral occipital cortex, and regions in the left and right middle frontal gyrus.

Connectivity analysis was performed for the intense-emotion task using anatomical regions defined from the Harvard-Oxford subcortical atlas (Desikan et al., 2006, Frazier et al., 2005, Goldstein et al., 2007, Makris et al., 2006), the sub-thalamic nucleus atlas (Forstmann et al., 2012) and the mean cortical activation of all subjects from the emotional images greater then neutral images contrast (Z > 2.3, p < 0.05 corrected and regions > 20 voxels). The anatomical regions include the anterior cingulate gyrus, posterior cingulate gyrus and left and right regions of the amygdala, thalamus, caudate, putamen, and subthalamic nuclei. The regions from the group activation of the intense-emotion task include regions in the left cerebral cortex, right frontal pole, right postcentral gyrus, left precuneus cortex and right superior frontal gyrus along with two regions in the left lateral occipital cortex, two regions in the left cingulate gyrus, three regions in the right lateral occipital cortex, and regions in the left and right middle frontal gyrus (Fig. 1). The average time course in each seed region was extracted and correlated with the average time course of all other seeds. The correlation coefficients were then converted to Fisher Z scores and used in a GLM analysis to look at differences in functional connectivity between the three groups. For each group comparison, all p values from all seed-seed correlations were corrected for multiple comparisons using the False Discovery Rate (FDR) correction (Benjamini and Hochberg, 1995).

### 2.9 Volumetric analysis

Subcortical volumetric analysis was performed using the subcortical segmentation of each participant's anatomical image (described in Section 2.6). The volume of each region was extracted from the segmentation and compared across the groups using a two-sample *t*-test and corrected for multiple comparisons using FDR correction. A comparison of cortical gray matter volume was implemented using FSL-VBM (Douaud et al., 2007, Good et al., 2001). The standard FSL VBM pipeline was used which included gray matter segmentation and non-linear registration (Andersson, 2007) in order to create a study-specific gray matter template that is aligned to the MNI 152 template. Each participants' gray matter segmentation was then non-linearly registered to the template and corrected for local expansion (or contraction) due to the non-linear component of the spatial transformation. The gray matter images were then smoothed with a 3 mm (sigma) isotropic Gaussian kernel. Differences in gray matter volume between the groups was computed using a voxel-wise GLM with non-parametric permutation testing with TFCE and 5000 iterations to correct for multiple comparisons.

## 3 Results

### 3.1 Demographics

Twenty-seven subjects with FT (age: 50.9 ± 12.0 years, 23 women, handedness: 1 left, 25 right, 1 ambidextrous) were matched to 25 HCs (48.6 ± 11.4 years, 21 women, handedness: 1 left, 23 right) but not, due to anticipated differences in disease demographics, to 16 subjects with ET (61.7 ± 9.3 years, 5 women, handedness: 1 left, 15 right, 3 unknown) (Table 1). Shorter disease duration in FT compared to ET subjects was expected (4.9 ± 6.0 years [range, 0.5 to 21] vs. 29.5 ± 16.3 years [range, 1.5 to 53]).

**Table 1:** Clinical features of study subjects.

	Subject/sex	Age (years)	Affected body part	Duration (years)	HAM-A	HAM-D	MINI	Medications
Functional tremor	1 F	52	BUE, BLE	18	15	17	MDD (P)	Duloxetine, alprazolam, primidone, amitriptyline, propranolol
	2 F	50	BUE	2	17	17	None	Milnacipran
	3 F	46	LUE, head	11	8	15	NGSP, OCD (C)	Clonazepam
	4 F	56	BUE	3	27	17	None	Duloxetine, modafinil, methylphenidate, imipramine, lorazepam, gabapentin
	5 M	40	LUE > RUE	20	6	19	None	Carbamazepine, citalopram
	6 F	64	RUE	11	0	5	MDD (C)	None
	7 F	49	BUE, BLE	21	15	19	MDD, PD-Ag, GSP, OCD, PTSD, GAD (C)	Risperidone, clonazepam, propranolol
	8 F	60	BUE	3	3	2	None	Lorazepam, valproate, sertraline
	9 F	34	BUE, head	3	2	16	PD-Ag, GSP, OCD, PTSD, Hypomania (C)	Duloxetine
	10 F	55	BUE	4	20	11	Agoraphobia (C)	Duloxetine, trazodone, alprazolam
	11 F	63	Head, trunk, BLE	3.5	1	3	None	None
	12 F	45	BUE	1	3	5	None	None
	13 F	58	RUE, stuttering	2	28	20	MDD, PD, PTSD, MDSP (C)	Citalopram, quetiapine, clonazepam, lamotrigine, sertraline
	14 F	38	RUE, RLE, eye	4	7	10	None	Citalopram, diazepam, propranolol, topiramate
	15 F	38	BUE, head	4	28	26	MDD, PD-Ag, PTSD, GAD (C)	Cyclobenzaprine, duloxetine
	16 F	38	BUE	1.2	3	3	None	Clonazepam
	17 F	43	BLE, stuttering	6	46	43	MDD, GAD, PD, PTSD (C)	Paroxetine
	18 M	27	RUE	1	0	0	None	Amitriptyline
	19 M	44	BLE > BUE, head	3.5	24	18	PD, PTSD (P)	Gabapentin, fluoxetine, clonazepam, baclofen
	20 F	66	LUE, BLE	1	22	11	None	None
	21 M	65	Head, RUE, LUE	1.5	5	7	None	None
	22 F	49	BUE	1	27	29	MDD, PD-Ag, PTSD (C)	Clonazepam, diazepam, venlafaxine
	23 F	52	BUE, BLE	0.5	23	32	MDD, GSP (C)	Valproate, gabapentin, alprazolam, baclofen
	24 F	75	RUE, RLE	1	13	10	None	Escitalopram
	25 F	58	Head, BUE	1.5	5	9	None	Primidone, propanolol, trazodone
	26 F	63	RUE, RLE	2.5	14	15	PD-Ag, OCD (C)	None
	27 F	53	Face, head, BUE, BLE	1	32	23	MDD, PD, GSP (C) alcohol dependence (P)	Clonazepam, desvenlafaxine, lamotrigine, lithium carbonate
Essential tremor	1	68	BUE	9	0	1	None	Propranolol, hydrocodone, ropinirole
	2	46	BUE	30	0	0	None	Propranolol, bupropion, duloxetine
	3	56	BUE	40	0	0	None	Atenolol
	4	56	RUE	1.5	5	9	None	Zolpidem, propranolol, amitriptyline
	5	47	BUE	40	0	1	None	Amitriptyline, primidone
	6	64	RUE > LUE	53	0	0	None	Primidone, gabapentin, propranolol, cyclobenzaprine
	7	55	BUE, neck	35	23	20	MDD, PD	Propranolol, quetiapine, lorazepam, lamotrigine, desvenlafaxine, flurazepam
	8	64	BUE	20	0	0	None	Unknown
	9	61	BUE	49	7	3	None	Citalopram, primidone
	10	74	BUE	14	4	4	None	Diazepam
	11	75	BUE, voice	20	5	2	None	Citalopram, clonazepam, primidone, topiramate
	12	50	LUE	20	7	4	GAD	Escitalopram, trazodone
	13	61	BUE	21	0	0	None	Propranolol
	14	69	BUE	50	0	1	None	Zolpidem
	15	73	BUE, head	20	0	0	None	Propranolol
	16	68	LUE, head	45	0	0	None	Unknown

HAM-D: 17-item Hamilton Depression Rating Scale; HAM-A: 14-item Hamilton Anxiety Rating Scale; MINI: Mini International Neuropsychiatric Interview to screen for axis I DSM-IV and ICD-10 psychiatric disorders; Ag: agoraphobia; OCD: obsessive-compulsive disorder; GAD: generalized anxiety disorder; GSP: generalized social phobia; MDD: major depression; MDSP: Mood Disorder with psychotic features; NGSP: non-generalized social phobia; PD: panic disorder; PTSD: post-traumatic stress disorder. RUE: right upper extremity; LUE: left upper extremity; RLE: right lower extremity; LLE: left lower extremity; RH: right hand; LH: left hand; RF: right foot; LF: left foot; B, bilateral; (C): current; (P): past.

### 3.2 Psychiatric features

Depression (HAM-D, 14.9 ± 10.1 vs. 2.8 ± 5.2 [HAM-D score 14–18 = moderate depression]) and anxiety scores (HAM-A, 14.6 ± 11.7 vs. 3.2 ± 5.9 [HAM-A score 14–17 = mild anxiety]) were significantly higher in the FT group compared to ET. Healthy controls had normal and low scores (HAM-D, 0.9 ± 1.6; HAM-A, 0.9 ± 2.2).

### 3.3 Motor processing: finger-tapping task

**Fig. 2:**
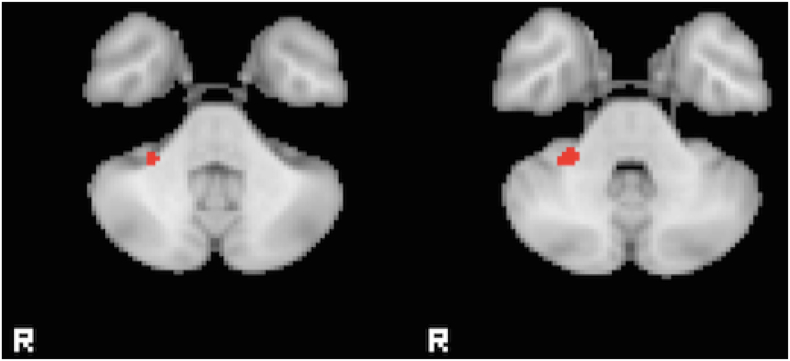
Differences in fMRI activation for the finger-tapping task. A region in the right cerebellum with increased activation in the FT group compared to ET during right tapping compared to rest. Images are shown at a threshold of Z > 2.3 and p < 0.05 corrected, after controlling for HAM-D.

Two small regions in the left precentral gyrus were found to have reduced activation in FT patients when compared with HC in the right tap greater than left tap contrast (Z > 2.3, p < 0.05 corrected), but this difference did not pass correction for multiple comparison when controlling for HAM-D. When controlling for HAM-D, there remained a significant reduction in the right cerebellum lobule VI in ET compared to FT for the right tapping greater than rest contrast (Fig. 2). No differences were found in the left tapping versus rest contrast. Average head motion for each group, as measured using the relative RMS displacement at a 50 mm radius (Power et al., 2012) was 0.11 ± 0.06 mm HC, 0.17 ± 0.18 FT, and 0.11 ± 0.04 ET with no significant differences between them.

### 3.4 Basic-emotion processing

**Fig. 3:**
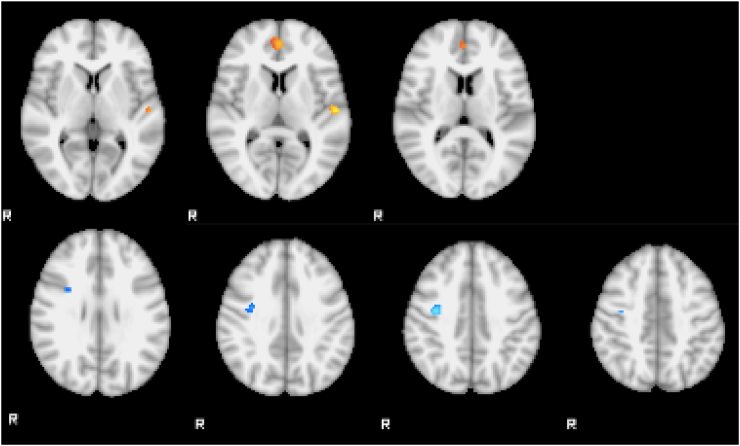
Differences in fMRI activation for the basic-emotion task (sad greater than neutral faces). Above, paracingulate gyrus and left Heschl's gyrus showed greater activation in the FT group than HC. Below, right precentral gyrus showed reduced activation in the FT compared to ET. Images are shown at a threshold of Z > 2.3 and p < 0.05 corrected, controlling for HAM-D.

In the sad faces compared to neutral faces, regional differences were found between the FT vs. ET groups and FT vs. HC groups when controlling for HAM-D. FT patients showed increased activation in the paracingulate gyrus and left Heschl's gyrus compared with HC and decreased activation in two regions in right precentral gyrus when compared with ET (Z > 2.3, p < 0.05 corrected) (Fig. 3). There were no significant differences between groups in the other contrasts. Average head motion for each group was 0.12 ± 0.04 mm HC, 0.16 ± 0.14 FT, and 0.09 ± 0.04 ET with no significant differences between them.

### 3.5 Intense-emotion processing

No differences between the groups during the intense-emotion processing task passed multiple comparison correction.

### 3.6 Connectivity analysis

A complete list of differences in pairwise connectivity between groups corrected at p < 0.05, and only significant for the intense-emotion task, is included in Table 2. When controlling for HAM-D, only the increase in connectivity in the FT group relative to HC between the left amygdala and left middle frontal gyrus survives the FDR correction.

**Table 2:** Summary of connectivity.

Contrast	ROI 1	ROI 2	t value	p value	FDR p
FT > HC	L putamen	R thalamus	3.566485696	0.000697516	0.034709915
	L putamen	L precuneus cortex	3.620004807	0.000588311	0.034709915
	L putamen	Post. cingulate G	4.003263873	0.000167205	0.034709915
	R middle frontal G	R postcentral G	3.544564582	0.000747598	0.034709915
	R occipital cortex	R occipital cortex 2	3.742310385	0.000396638	0.034709915
	R putamen	L precuneus cortex	3.410815902	0.001135415	0.043372047
	L amygdala	L middle frontal G	3.337452006	0.001422406	0.046228209
	Ant. cingulate G	L middle frontal G	3.262964607	0.001782948	0.048288185
	Post. cingulate G	R putamen	3.267808005	0.001757106	0.048288185
HC > FT	L putamen	R putamen	3.392599168	0.001201072	0.043372047
	L putamen	R superior frontal G	3.649168746	0.00053587	0.034709915
	L caudate	R superior frontal G	3.661756816	0.00051464	0.034709915
ET > FT	R thalamus	R superior frontal G	4.940914954	6.04E-06	0.001964324
FT > HC (HAM-D regressed)	L amygdala	L middle frontal G	4.03383398	0.000152953	0.049709585

HC: healthy controls; FT: finger tapping; ET: essential tremor; G; gyrus; ROI: region of interest; FDR: false discovery rate.

### 3.7 Volumetric analysis

Compared to HC, patients with FT showed a smaller volume in the left caudate (corrected p < 0.022). Patients with ET showed a larger volume of the right amygdala compared to patients with FT and HCs (corrected p < 0.001 and p < 0.032, respectively). The VBM analysis showed a small region of reduced gray matter in the right postcentral gyrus in FT patients when compared with controls. It also showed reduced gray matter in ET in the left and right cerebellum (VIII) when compared with FT and left cerebellum (VII) and left occipital pole when compared with HC.

## 4 Discussion

Our main findings are that, after controlling for depression scores and correction for multiple comparisons, FT is associated with increased activation in the right cerebellum compared to ET during the motor task; and increased activation in the paracingulate gyrus and left Heschl's gyrus compared with HC along with decreased activation in the right precentral gyrus when compared with ET during the basic-emotion task (sad faces greater then neutral faces). While there were no significant differences during the intense-emotion task FT subjects exhibited an increase in connectivity between the left amygdala and the left middle frontal gyrus during this task as compared to HC. Collectively, these findings suggest that patients with FT exhibit altered activation of key cognitive and limbic structures in a pattern that is distinct from ET and HC.

This is the first study probing emotional circuitry specifically in patients with FT. Voon and colleagues examined the brain activation patterns using fMRI and a simple facial emotion paradigm in 16 patients with a variety of functional disorders (10 had tremor) and found no group differences on primary analysis compared to HCs even after controlling for concurrent depression and anxiety (Voon et al., 2010). However, they identified greater right amygdala activity during happy stimuli on post-hoc analysis in the functional group. The study did not assess differences between simple and intense emotion processing paradigms, which limit comparisons with ours. More recently, Aybek and colleagues evaluated fMRI changes to stimuli of faces with fearful and sad emotional expressions in comparison to faces with neutral expressions in 12 patients with a variety of neuropsychiatric symptoms qualifying as conversion disorder and found increased amygdala activation to negative emotions compared to healthy controls (Aybek et al., 2015). Using a larger sample and more homogenous functional phenotype, we identified the left amygdala as an area of abnormally increased connectivity (with the middle frontal gyrus) during intense stimuli. Importantly, when correcting for depression, which was more common in the FT cohort, many abnormalities on activation patterns disappeared, suggesting a strong influence of mood states on the neurobiological effects of emotional stimuli. Our study was not designed, however, to properly assess such effect since matching for the presence of major depression, the most common psychiatric comorbidity, was not performed (the MINI screen uncovered major depression in one third of FT patients but in only 2 ET patients).

Several pathophysiologic mechanisms can be entertained. On one level, the differences in connectivity by the amygdala and, therefore, the limbic system and striatum, suggests that abnormal processing of emotional information is associated with limbic overactivation (Harvey et al., 2006). Emotional stimuli may thus affect somatosensory processing by limbic areas associated with emotion and attention (Black et al., 2004). On the other hand, increased activation in the paracingulate gyrus, which is associated with the anterior cingulate cortex, may represent in FT subjects the neurobiological correlate of alexithymia, the inability to identify and describe emotions (Kaplan et al., 2013). Indeed, high alexithymia scores have been shown to increase activity in the anterior cingulate, mediofrontal cortices, and insula during emotional stimuli processing (Deng et al., 2013), a pattern similar to ours. Finally, increased activation of the paracingulate gyrus may represent a compensatory neural mechanism during emotional processing, modulating emotional responsiveness via top-down cognitive control, as recently shown in patients with Parkinson disease (Moonen et al., 2017).

The anterior paracingulate cortex is considered a key prefrontal region subserving the theory of mind, the ability to represent mental states, which is important in understand the intentions of people involved in social interaction and in predicting future intentional social interaction (Walter et al., 2004). The impaired reasoning about our own and other people's mental states has already been identified in another functional disorder, psychogenic non-epileptic seizures (also with increased alexithymic traits) (Schonenberg et al., 2015). Theory of mind can provide a unifying vision of functional neurological disorders given that it may serve to form a representation of mental states by reconciling the perception of the internal states of the body, or interoception, across a variety of emotional states (Shah et al., 2017). In the setting of altered emotion processing (possibly with alexithymia), the ability to predict future events as desired is eroded (Bartsch and Estes, 2014). This impaired “Bayesian prediction” may explain the ostensible contradictions between voluntary and unconscious motor control in FMD, with abnormal predictive beliefs generating movements executed without a sense of agency (Newby et al., 2016).

Our study has limitations. Although it was powered to detect major differences in activation by emotion processing tasks, its relatively small sample precludes an adequate assessment of the effect of psychiatric co-morbidities (such as depression, PTSD, and anxiety) on the activation patterns in the motor and emotional tasks. It is possible that these comorbidities may have contributed to at least some of the differences in activation patterns identified in FT subjects, suggesting maladaptive cognitive interpretation of emotions (Harrison and Critchley, 2007). Similarly, due to sample size, we are unable to determine whether sidedness and topographical involvement (e.g., arm versus neck involvement) may be contributory. Nevertheless, the variance in phenomenological involvement, characteristic of the referral pattern of a specialized movement disorders center, would have diluted the statistical differences between groups rather than create false positive findings. In addition, we did not capture resting-state fMRI data and our findings cannot therefore be compared to those recently reported by Maurer and colleagues demonstrating decreased functional connectivity between the right temporo-parietal junction and bilateral sensorimotor regions (Maurer et al., 2016). Finally, we did not measure alexithymia in our patients, which has been recently shown to contribute to the development of functional disorders (Demartini et al., 2014) and to impaired facial emotion recognition in these patients (Pedrosa Gil et al., 2009), and may have mediated at least some of the cortical activation in response to emotion stimuli.

## 5 Conclusions

Functional tremor is associated with abnormalities in activation and functional connectivity in networks involved in emotion processing and theory of mind, which may be relevant to the pathophysiology of functional movement disorders. Prospective studies will be needed to determine whether the observed changes are relevant to the pathogenesis of functional disorders or may represent changes that are compensatory or secondary to primary motor or psychological states. It will also be important to examine the extent to which promising interventions, such as cognitive behavioral therapy (LaFrance et al., 2014), are capable of normalizing the abnormalities in emotion processing identified in this study.
